# Oxidized mannan-MOG35-55 conjugate as a platform for antigen-specific immune modulation in multiple sclerosis

**DOI:** 10.3389/fimmu.2026.1809907

**Published:** 2026-04-28

**Authors:** Athanasia Mouzaki, Anne-Lise de Lastic, Vasso Apostolopoulos, Ioannis Matsoukas

**Affiliations:** 1 Laboratory of Immunohematology, Division of Hematology, Medical School, University of Patras, Patras, Greece; 2 School of Health and Biomedical Sciences, RMIT University, Melbourne, VIC, Australia; 3 NewDrug, P.C., Patras Science Park, Patras, Greece

**Keywords:** mannan conjugate, MOG35-55, multiple sclerosis, myelin, peptides, personalized immunotherapy, regulatory T cells

## Abstract

Multiple sclerosis (MS) is a chronic autoimmune disease of the central nervous system (CNS) characterized by autoreactive T-cell responses against myelin antigens. Current disease-modifying therapies (DMTs) broadly suppress immune activity but do not restore antigen-specific immune tolerance and may cause significant adverse effects. Antigen-specific immunotherapies offer a rational alternative aimed at selectively re-educating the immune system without compromising protective immunity. Structural determinants of myelin antigens, including arginine-dependent conformational stability and post-translational modifications such as citrullination, critically influence their immunogenic and tolerogenic properties. Recent advances highlight the potential of oxidized mannan-conjugated myelin oligodendrocyte glycoprotein peptide (OM-MOG35-55) to modulate antigen-specific immune responses in experimental models and human immune cells. Studies suggest that OM-MOG35-55 can influence dendritic cell (DC)-mediated antigen presentation and favor the expansion of CD4+PD-1+ and CD4+CD25+Foxp3+ T-cell populations and the production of regulatory cytokines such as IL-10 and TGF-β1. When combined with vitamin D3 conditioning of DCs, the immunomodulatory potential of OM-MOG35-55 appears enhanced. Nevertheless, the precise mechanisms underlying OM-MOG-mediated immune modulation are not fully defined, and the antigenic heterogeneity of MS and the limited predictive value of EAE models highlight the need for cautious interpretation of preclinical findings. This mini-review integrates structural, immunological, and translational evidence supporting OM-MOG35-55 as a promising platform for antigen-specific immunotherapeutic design and proposes a roadmap toward clinical validation of this immunotherapeutic strategy for MS.

## Introduction

MS is a chronic autoimmune demyelinating disease of the CNS in which autoreactive T lymphocytes and antibodies target myelin components, leading to inflammation, demyelination, axonal damage, and progressive neurological disability (1, 2). Additionally, regulatory T cells (Tregs), particularly CD4+CD25+Foxp3+ natural Tregs, which normally maintain immune tolerance in autoimmune settings, are numerically and functionally impaired in MS patients (1–3). Autoreactive CD4+ T helper (Th) cells, especially Th1 and Th17 subsets, recognize epitopes of myelin proteins including myelin basic protein (MBP), proteolipid protein (PLP), and myelin oligodendrocyte glycoprotein (MOG).

MS is immunologically heterogeneous, and different patients may exhibit autoreactivity toward distinct myelin epitopes. Therefore, immunodominant targets identified in animal models of MS (experimental autoimmune encephalomyelitis, EAE), such as MOG35-55, may not represent the full spectrum of antigens driving human disease. This heterogeneity presents a major challenge for antigen-specific therapeutic approaches and highlights the need to establish the reactivity of patients’ immune cells to antigenic epitopes to design personalized mono-epitope or multi-epitope strategies (4).

Current DMTs reduce relapse rates and delay disease progression but act through broad immunosuppression or immune deviation and do not restore antigen-specific tolerance. Long-term treatment may be associated with infections, malignancies, and other adverse effects (1). These limitations have driven sustained interest in antigen-specific immunotherapies designed to selectively silence pathogenic immune responses while preserving global immune competence (5).

Early clinical trials using peptides derived from MBP or PLP showed that modulation of peptide structure can profoundly affect T-cell responses, but these approaches yielded mixed efficacy and, in some cases, unexpected immune activation or hypersensitivity reactions (6–9). These outcomes highlighted the need for antigen-delivery platforms that actively promote immune regulation rather than simply altering T-cell receptor (TCR) signaling strength.

Beyond antigen identity, the structural integrity of myelin proteins plays a decisive role in immune recognition. Arginine residues are critical for maintaining the native conformation of MBP and MOG through electrostatic interactions. Post-translational modification of arginine to citrulline, catalyzed by peptidylarginine deiminases (PADs), neutralizes positive charge, destabilizes protein folding, and alters peptide-MHC class II interactions (10–14). Extensive citrullination of MBP has been documented in MS patients and is associated with enhanced Th1 polarization and increased production of pathogenic cytokines (13). Experimental studies further demonstrate that substitution of key arginine residues within the MOG35-55 epitope attenuates disease severity in experimental autoimmune encephalomyelitis (EAE), underscoring the importance of arginine-dependent structure in disease pathogenesis (15).

These observations provide a strong mechanistic rationale for antigen-specific tolerance strategies that not only target immunodominant epitopes but also override pathogenic structure-driven immune activation. Mannan conjugation of MOG35-55 represents such a strategy, redirecting antigen presentation toward regulatory immune pathways rather than inflammatory responses.

## Mannan-MOG35-55: A next-generation tolerogenic platform

Mannan is a polysaccharide derived from yeast cell walls that interacts with pattern-recognition receptors including the mannose receptor and Toll-like receptor 4 (TLR4) on DCs, thereby influencing antigen uptake, processing, and signaling pathways (16).

Experimental studies indicate that conjugating mannan to antigenic peptides enhances antigen presentation and modulates dendritic-cell activation pathways. In EAE models, MOG35-55 conjugated with oxidized mannan (OM-MOG35-55) suppresses disease onset and progression in both prophylactic and therapeutic settings. These effects are antigen-specific, associated with reduced pathogenic T-cell proliferation, and occur without global immunosuppression. Notably, differences have been observed between oxidized and reduced mannan (RM) conjugates depending on the experimental context. In prophylactic vaccination, OM-MOG35-55 appears more effective than RM-MOG35-55, whereas in therapeutic administration after disease induction, both forms display comparable efficacy (17, 18). Furthermore, strain-specific differences have been reported. For example, OM-MOG35-55 vaccination was effective in C57BL/6 models but not in SJL/J mice, while OM-PLP139–151 showed the opposite pattern; in SJL/J mice, RM-MBP83–99 yielded the best immunological profile (19). These findings suggest that the type of antigen and host genetic background strongly influence therapeutic outcomes.

Importantly, OM-MOG35-55 remains structurally stable and resistant to enzymatic degradation, and can be analytically detected after conjugation (20, 21), properties essential for consistent immunological activity. The protective efficacy of OM-MOG35-55 has been validated in HLA-DR2 transgenic mice, supporting its translational relevance to human MS (17).

Structure-activity relationship studies further demonstrate that the integrity of conserved arginine residues within MOG35-55 is critical for modulating immune responses. Substitution of Arg41 and Arg46 with alanine produces analogues that significantly reduce the severity of MOG-induced EAE, illustrating how subtle structural changes influence pathogenicity and tolerance induction (15).

## Translation to the human immune system

Building on animal studies, the effects of OM-MOG35-55 have been investigated in human immune cells. In a previous study using peripheral blood mononuclear cells (PBMCs) from 83 patients with relapsing-remitting MS (RRMS) and 45 healthy controls, both the frequency and suppressive function of CD4+CD25+Foxp3+ Tregs were found to be significantly reduced during active disease, whereas exposure to myelin antigens in culture resulted in modest changes in Treg frequency and suppressive activity (3). Notably, in this experimental setting, culture with OM-MOG35-55 induced the highest suppressive activity of Tregs, as assessed by measuring anti-inflammatory cytokines in culture supernatants, compared with other peptides used (3). This study indicated tolerance can be restored through CD4+CD25+Foxp3+ Treg induction with OM-MOG35-55 and set the stage for subsequent experiments designed to simulate the *in vivo* situation in humans. The results of these experiments demonstrated that DCs differentiated from patient-derived monocytes and pulsed with OM-MOG35-55 induced strong regulatory responses after repeated antigen-presentation cycles (22). Antigen presentation favored memory CD4+ T-cell populations with reduced effector activation, while markedly expanding CD4+PD-1+ T cells and CD4+CD25+Foxp3+ Tregs. The cytokine environment was reprogrammed toward tolerance, with increased secretion of TGF-β1 and IL-10 and suppression of IL-6, TNF-α, and IL-17 (22).

Vitamin D3 conditioning of DCs further enhanced these tolerogenic effects (22), most likely by modulating cellular metabolism and epigenetic programs that stabilize regulatory T-cell differentiation, such as chromatin accessibility, regulation of transcriptional programs controlling regulatory cytokine production and metabolic pathways influencing tolerogenic dendritic cell function (23, 24).

It is noteworthy that the patients and controls who donated blood for the OM-MOG35-55 studies with human PBMCs (3, 22) were not HLA-typed, yet cells isolated from all blood samples responded to the peptides. The activity of OM-MOG35-55 across individuals with different HLA phenotypes may be explained by the ability of class II-restricted peptides to be presented by multiple HLA molecules. Although HLA class II molecules are highly polymorphic, many peptides display promiscuous binding and can be presented by more than one HLA-DR or HLA-DQ allele. This phenomenon is well documented in the class II literature, where shared anchor residues enable peptide binding across different HLA-DR molecules (25, 26).

Regarding myelin antigens, MOG-derived peptides have been shown to be recognized in various HLA contexts. *In vitro* assays with MOG35-55 demonstrated that this peptide can bind to HLA-DR2 with high affinity (27). Additionally, MOG T-cell epitopes restricted by HLA-DRB1*0401 were identified in HLA-DR4 transgenic mice and shown to be presented by human antigen-presenting cells (28). Previous work using mannan-conjugated MOG35-55 also demonstrated peptide-specific tolerogenic activity in experimental autoimmune encephalomyelitis models, including humanized HLA-DR2b mice, supporting the concept that the immunomodulatory effects of this peptide are not limited to a single MHC class II background (18).

Human PBMC assays also reflect heterogeneous antigen-presenting and T-cell repertoires from donors with diverse HLA genotypes. Therefore, the responses observed *in vitro* likely reflect modulation of the responding cellular repertoire rather than a single peptide-HLA restriction pair. In this context, presentation of the peptide by multiple class II molecules may be sufficient to engage and tolerize or anergize autoreactive T-cell populations. Consistent with this interpretation, antigen-specific tolerance strategies in autoimmune disease often operate through induction of regulatory or anergic pathways within antigen-responsive T-cell pools, including mechanisms involving IL-10-producing regulatory T cells and tolerogenic antigen presentation (29–31).

## Mechanistic integration: structure, folding, and immune regulation

The tolerogenic efficacy of OM-MOG35-55 results from the convergence of structural and immunological mechanisms. Arginine-dependent conformational stability of myelin proteins determines baseline immunogenicity, while citrullination-driven misfolding promotes pathogenic Th1-biased responses (11, 13, 14). Mannan conjugation functionally overrides these pro-inflammatory cues by reprogramming antigen presentation at the level of DCs, leading to durable regulatory T-cell induction (22).

Previous studies showed that citrullination of arginine residues within MBP peptides enhances Th1 polarization in PBMC cultures and exacerbates autoimmune responses (10, 13, 32). In contrast, rational design of altered MOG peptides and conjugation of myelin antigens to oxidized or reduced mannan through defined peptide linkers have produced constructs with favorable immunomodulatory profiles in EAE models and human cells (17, 18, 22).

The mannan conjugation strategy has also been successfully applied by using the MBP83–99 immunodominant epitope conjugated to mannan via the (KG)5 bridge (19).

Additionally, an alternative strategy based on the MOG35-55 antigen is cyclisation of the linear sequence, which led to greater amelioration of clinical and neuropathological features of EAE (27).

## Personalized immunotherapy framework

Mannan conjugates have already been used in human cancer immunotherapy, demonstrating an excellent safety profile (33).

**Figure 1:**
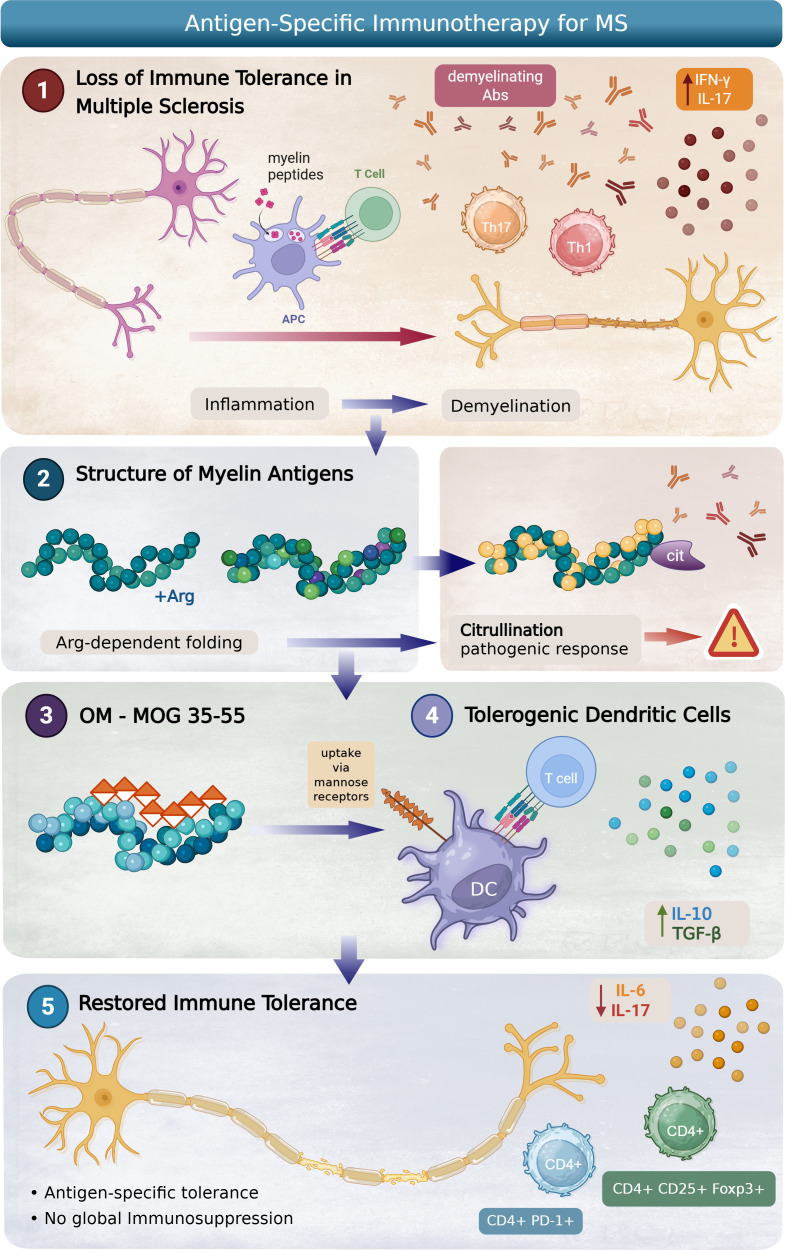
Antigen-specific immunotherapy strategy for restoring immune tolerance in multiple sclerosis (MS). (1) Loss of immune tolerance in MS. Myelin-derived peptides are presented by antigen-presenting cells (APCs) to autoreactive T cells, promoting their differentiation into pro-inflammatory Th1 and Th17 cells. This response is associated with the production of inflammatory cytokines such as IFN-γ and IL-17, as well as demyelinating antibodies, leading to neuroinflammation and myelin damage. (2) Structure and modification of myelin antigens. Myelin antigens exhibit arginine-dependent folding. Post-translational modifications such as citrullination alter antigen structure and enhance pathogenic immune recognition, contributing to disease progression. (3) Engineered myelin oligodendrocyte glycoprotein (OM-MOG 35-55). A modified MOG peptide is designed for targeted delivery and uptake via mannose receptors on dendritic cells (DCs), facilitating antigen-specific immune modulation. (4) Induction of tolerogenic DCs. Antigen-loaded DCs adopt a tolerogenic phenotype, interacting with T cells to promote anti-inflammatory signaling and the release of regulatory cytokines such as IL-10 and TGF-β. (5) Restoration of immune tolerance. This approach induces antigen-specific immune tolerance without global immunosuppression, characterized by reduced pro-inflammatory cytokines such as IL-6 and IL-17 and expansion of regulatory T cell populations (CD4+PD-1+ and CD4+CD25+ Foxp3+), ultimately supporting remyelination and neural repair. Figure created using BioRender (https://biorender.com).

OM-MOG35-55 reflects several principles of precision immunotherapy: (i) antigen specificity, by targeting an immunodominant epitope implicated in MS pathogenesis; (ii) structural insight, by preserving or functionally redirecting arginine-dependent conformations; (iii) autologous implementation, using patient-derived DCs compatible with individual HLA backgrounds; and (iv) adjunct modulation, through clinically accessible vitamin D supplementation. This integrated framework offers the prospect of individualized, disease-modifying immunotherapy capable of resetting immune tolerance without compromising host defense (**Figure 1**).

Antigen-specific tolerogenic strategies have entered early-phase clinical testing in MS, including peptide-loaded tolerogenic dendritic-cell approaches, supporting the translational relevance of this therapeutic direction (31).

## Conclusions and future perspectives

Structure-guided mannan conjugation of MOG35-55 provides a robust and mechanistically grounded approach to antigen-specific immunotherapy in multiple sclerosis. By integrating insights into arginine-dependent protein structure, citrullination-driven pathogenicity, and DC-mediated immune regulation, OM-MOG35-55 provides a rational foundation for personalized tolerance-inducing therapies capable of modifying disease course without compromising systemic immunity.

However, given the multi-antigen nature of MS, several key questions should be addressed to advance OM-MOG35-55 toward clinical application:

- Whether OM-MOG primarily induces Treg differentiation and/or promotes T-cell anergy or exhaustion.
- Whether it enables bystander suppression.
- Longitudinal tracking of TCR repertoires and stability of induced Tregs.
- Assessment of whether OM-MOG35-55 has better effects in patients with high anti-MOG antibody titers compared to those patients with control-level anti-MOG antibody titers (34).
- Optimization of dosing intervals and administration routes (ex-vivo DC loading versus direct peptide delivery).
- Evaluation of OM-MOG35-55 in progressive and secondary MS subtypes.
- Assessment of OM-MOG35-55 as monotherapy or add-on therapy.

**Table 1** summarizes the current landscape of antigen-specific immunotherapeutic approaches in MS, highlighting key strategies, their underlying mechanisms, experimental systems, and principal limitations. This comparative framework places OM-MOG35-55 within the broader context of the field, emphasizing the context-dependent efficacy of different platforms, and underscores the challenges associated with translating antigen-specific tolerance strategies into clinical application.

**Table 1:** Antigen-specific immunotherapeutic strategies in MS.

Strategy/Platform	Antigen(s)	Model/System	Key immunological effect	Limitations/Considerations	Key refs.
OM-MOG35-55	MOG35-55	EAE (C57BL/6), HLA-DR2 mice; human ex-vivo (PBMCs and DCs)	Induces tolerogenic DCs; promotes CD4+CD25+Foxp3+ Tregs and CD4+PD-1+ T cells; increases IL-10 and TGF-β1; suppresses IL-6, TNF-α, IL-17	Antigen- and strain-dependent efficacy; limited clinical validation; MS heterogeneity may limit universal applicability	(17, 18, 22)
RM-MOG35-55	MOG35-55	EAE (therapeutic setting)	Comparable immunomodulatory efficacy to OM-MOG35-55 in therapeutic administration	Less effective in prophylactic settings; context-dependent performance	(17, 18)
OM-PLP139-151	PLP139-151	EAE (SJL/J mice)	Effective modulation in PLP-driven disease models	Opposite strain specificity compared to MOG; highlights antigen dependence	(19)
RM-MBP83-99	MBP83-99	EAE (SJL/J mice)	Strong immunomodulatory profile in MBP-driven EAE	Limited cross-model generalizability	(19)
Mannan-conjugated MBP peptide	MBP83-99	EAE	Antigen-specific immune modulation using linker-based conjugation ((KG)_5_ bridge)	Platform-specific optimization required	(19)
Free peptide/altered peptide ligands	MBP, PLP peptides	Human clinical trials; EAE	Modulation of T-cell responses via altered TCR signaling	Mixed efficacy; risk of hypersensitivity or disease exacerbation	(6–9)
Tolerogenic DCs (tolDCs)	Myelin peptides (e.g., MOG35-55)	Human ex vivo; Phase 1b clinical trial	Induce antigen-specific tolerance; expand Tregs; suppress autoreactive T cells	Complex manufacturing; inter-patient variability; scalability challenges	(22, 31)
Vitamin D3-conditioned tolDCs	Myelin peptide-loaded DCs	Human ex vivo	Enhances tolerogenic DC function via metabolic and epigenetic reprogramming; increased IL-10 production	Mechanistic complexity; variability in donor response	(22–24)
Oral/peripheral tolerance strategies	Myelin peptides	EAE; preclinical	Induction of Tregs and immune tolerance	Often weak or transient; context-dependent efficacy	(29, 30)
Cyclized MOG peptides	Cyclic MOG35-55	EAE	Improved structural stability and enhanced therapeutic efficacy vs linear peptide	Still preclinical; unclear translation to humans	(27)
Multi-epitope/personalized antigen strategies	Multiple myelin epitopes	Conceptual/translational	Addresses antigenic heterogeneity of MS; enables personalized immunotherapy	Increased complexity; requires patient-specific profiling	(4, 5)

Several emerging strategies may complement antigen-specific tolerance induction. Chimeric antigen receptor (CAR) T-cell therapies, initially developed for oncology, are now being explored in neuroautoimmune diseases including MS, with early evidence suggesting that selective targeting of pathogenic immune populations may be feasible (35, 36).

At the same time, accumulating data implicate Epstein-Barr virus (EBV) infection as a major environmental contributor to MS risk. EBV reactivation is associated with altered immune regulation, and arginine-rich viral proteins such as ZEBRA may mechanistically intersect with myelin-directed autoimmunity (37–40).

Rather than representing competing paradigms, these approaches may ultimately converge with peptide-based tolerance platforms such as OM-MOG35-55 to form integrated therapeutic strategies that address both antigen-specific and upstream disease drivers.
